# Considerations for the Telehealth Systems of Tomorrow: An Analysis of Student Perceptions of Telehealth Technologies

**DOI:** 10.2196/mededu.5392

**Published:** 2016-07-08

**Authors:** Tyler Preston Bull, Alexis Roxanne Dewar, Donna M Malvey, James Leo Szalma

**Affiliations:** ^1^ University of Central Florida Department of Psychology College of Sciences Orlando, FL United States; ^2^ University of Central Florida Department of Health Management and Informatics College of Health and Public Affairs Orlando, FL United States

**Keywords:** telehealth systems, younger adults, telehealth advantages, telehealth disadvantages, thematic analysis

## Abstract

**Background:**

While much is known about factors that facilitate telehealth adoption, less is known about why adoption does or does not occur in specific populations, such as students.

**Objective:**

This study aims to examine the perceptions of telehealth systems within a large student sample.

**Methods:**

Undergraduate students (N=315) participated in a survey of the perceived advantages and disadvantages of telehealth technologies. The responses to the survey were analyzed using thematic analysis.

**Results:**

We found that students were likely to adopt telehealth systems for the following reasons: (1) the system worked efficiently, (2) the convenience of telehealth, and (3) to gain access to health services. Students also perceived several disadvantages to telehealth systems, such as issues of trust (ie, security, privacy), the impersonal nature of telehealth systems, and they were concerned about the potential for major system errors.

**Conclusion:**

By understanding the current barriers to telehealth adoption in a cohort of students, we can not only better anticipate the future needs of this group, but also incorporate such needs into the design of future telehealth systems.

## Introduction

Telehealth systems are integral to the exchange of electronic health care information between patients and providers. These systems have also vastly improved access to care, as well as the quality of care received [1-6]. Moreover, telehealth has significantly reduced the cost of health care in many countries [1,7-10]. In one meta-analysis, the impact of telemedicine on the management of chronic diseases (eg, diabetes, hypertension) was overwhelmingly positive [11]. Only two studies in this analysis of randomized control trials (RCTs) (N=148) reported negative effects of telehealth. Because of the success of telehealth technologies, the American Telemedicine Association projects that the usage of these systems is expected to double or triple within the next five years [1,12].

Several theories have aimed to explain the widespread adoption of telehealth technologies. One such theory includes the Health Belief Model (HBM), which suggests that perceived disease threat (PDT) and behavioral evaluation (PB) are key factors in telehealth acceptance [13]. PDT is an individual’s perception of the severity of an ailment and the perceived risk associated with that health condition, whereas PB encompasses the steps an individual takes to reduce the likelihood of a particular disease or illness. One drawback of the HBM is that it may only apply to home-based telehealth systems. In addition, there has been limited replication of this model within the telehealth literature and it is not widely used.

One theory that overcomes some of the issues with the HBM is the Technology Acceptance Model (TAM). The TAM suggests that the adoption of a telehealth system is broadly determined by its perceived usefulness (PU) and perceived ease of use (PEOU). PU and PEOU each consist of several subconstructs related to telehealth technology adoption, including motivation and behavioral intentions [14-16]. Many researchers have extended the TAM by incorporating user trust [17], technology readiness [18], or perceived threat (ie, technology will replace a job) [19]. Since the TAM was first introduced, it has been very successful in predicting telehealth acceptance and adoption (variance accounted for ranges from 50% to 70% in most studies) across many populations (eg, veterans, older adults, etc.) [14-16]. However, the TAM is not without its flaws. For example, more nuanced research on system trust needs to be conducted before this factor can be fully integrated into the TAM, as this literature has yielded mixed results [20,21]. Furthermore, the TAM does not address or incorporate the severity of illnesses or the impact of disease burden into its framework like the HBM. Rather, the TAM suggests that, regardless of disease, most individuals will adopt telehealth systems for reasons of perceived usefulness and usability.

### Perceptions of Telehealth Systems in Student Populations

To date and to the best of our knowledge, only one study has previously attempted to measure student readiness to adopt telehealth technologies. In this study, 308 undergraduate nursing students participated in an online survey about their anticipation of interacting with telehealth devices, such as telenursing tablets, telerobots, and teleconferencing [22]. In this survey, they found that 66% of respondents would definitely use a telehealth device in their future careers as nurses, and another 70% believed that telenursing should be incorporated into the educational curriculum. Many students indicated that they viewed telehealth technologies positively and saw these devices as having many advantages. However, specific advantages were not reported within this study.

### The Present Study

The goal of the present study is to examine student perceptions of the advantages and disadvantages associated with telehealth systems. By understanding the current barriers to telehealth adoption in a cohort of students, we can not only better anticipate the future needs of this group, but also incorporate such needs into the design of future telehealth systems. One approach to studying the perceived advantages and disadvantages of this cohort is to conduct a thematic analysis.

## Methods

### Thematic Analyses

A thematic analysis studies the themes, or subthemes, that emerge through open-ended survey items. This technique is used to detect trends in open-ended survey responses and results in a deeper, richer sense of the data. As best stated by Braun and Clarke, “thematic analysis is a useful and flexible method for qualitative research in and beyond psychology” (p2) [23]. With regard to the present study, we elected to use grounded theory, a technique that develops themes based on the pattern and frequency of particular responses [24,25].

First, 2 researchers independently identified themes in a randomized subset of the open-ended responses collected from our sample. The researchers then compared the themes they identified independently and collapsed them into 2 lists: 1 for perceived advantages and 1 for perceived disadvantages. The data was then rated by 2 researchers based on these lists of themes. A particular response could be rated as multiple themes if it contained elements from each of these types of themes (ie, the open-ended comment discussed themes about both usability and trust). The researchers were instructed to rate each individual’s response as containing as many themes as were relevant. If a particular comment did not fall into a theme on either list, it was not rated. The researchers were provided with definitions corresponding to each theme (Table 1).

After all of the responses were rated, Cohen’s Kappa (κ) was calculated as a measure of interrater reliability. Kappa statistics were calculated for perceived advantages (κ= .838) and perceived disadvantages (κ= .896). The agreement between the 2 raters was strong [26,27]. The raters agreed on the classification of the themes approximately 84% to 90% of the time.

### Procedures

The survey was administered online via an anonymous link using Qualtrics survey software. After electronically signing the informed consent, participants were asked to read our operationalization of telehealth technology [6]. This ensured that all participants were familiar with telehealth systems and that they could respond to all survey items bearing the entire definition in mind. The definition read as follows:

Telehealth is the exchange of medical information from one party to another via electronic communication. It is used to improve a patient’s clinical health and mental health status. Telehealth includes using two-way streaming video, email, smart phones, smart watches, wireless tools, or other forms of electronic telecommunications to interact with a medical professional.

6

The entire survey took approximately 30 minutes to complete. Data was collected from January 2015 until June 2015.

**Table 1:** Definitions of each theme.

Theme or subtheme		Definitions and examples
Advantages		
	Accessibility	Improved access to health services; access to health professionals
	Convenience	Data is stored on the device; readily access data; avoid excess travel
	Efficiency	Quick communication; rapid connection to services
	Affordability	Cost of telehealth is within price range; cost effective
	Anonymity	More disclosure of embarrassing or sensitive health information
	Communication	Better communication with provider; written record of conversation
	Connectedness	Improved relationship with health provider; closeness with provider
	Usability	System is designed well; intuitive; organized; modern interface
Disadvantages		
	Trust	Issues involving privacy and security of health information or data
	Impersonality	Fear of machines replacing health care professionals; less connectedness
	System errors	Fear of misdiagnosis; test results are not credible; loss of health information
	Communication	Greater chance of miscommunication; asynchronous response/feedback
	Affordability	Cost of telehealth is out of price range; not cost effective

### Measures

#### Demographics

Items related to student health status included items about mental health, chronic disease, and any other medical complications participants could be experiencing at the time of the survey. Since this information is very sensitive, participants were reminded that they did not have to respond to these questions if they felt uncomfortable. Additional demographic information (eg, age, gender, nationality, etc.) was collected at the end of the survey. Demographic items were administered at the end of the survey to reduce any possible cognitive bias (ie, mental health conditions are still perceived negatively and this could in turn effect how students with a mental illness respond to health-related items).

#### Open-Ended Survey Items

Two open-ended survey questions were asked during a larger replication study on health and technology. The first open-ended survey item was related to advantages. It read as follows: “Why would you use the telehealth device again in the future?” The second open-ended survey item was related to the perceived disadvantages of telehealth systems. It read as follows:

*Why would you not use the telehealth device again in the future? Do you have any concerns about interacting with a telehealth device again in the future?*

### Participants

To be eligible to participate in the survey, participants had to have interacted with a telehealth device within the past year and specify the name of said device. Participants were awarded course extra credit for completing the survey, which could be applied to a psychology course in which they were enrolled. All participation was voluntary. The University of Central Florida Institutional Review Board approved all procedures and materials used in this study.

In total, 315 undergraduate students (108 male; 206 female; 1 transgender) between the ages of 18 to 49, with a mean (SD) of 20.69 (4.03) years and a median of 19.00 years, met the above study criteria and were recruited from the psychology research participation system at the University of Central Florida. Of these 315 students, 295 (96.7%, 295/315) responded to the advantages open-ended item and 303 (96.2%, 303/315) responded to the disadvantages open-ended item, which were part of a larger study replication on health and anticipated technology usage (the results of this study are published elsewhere) [28]. The larger study consisted of 2 measures (40 items on health technology engagement; 26 items on the psychological impact of assistive devices), open-ended response questions, and participant demographics.

## Results

### Descriptive Statistics

Generally, our sample was healthy and reported exercising at least once per week for 30 to 60 minutes (on average). Most participants reported that they did not have a mental health concern arise within the past year. Few participants reported a chronic or acute medical condition. All demographic information is reported in Table 2.

**Table 2:** Participant demographics (N=315).

Item		Students, n (%)
Gender		
	Female	206 (65.3)
	Male	108 (34.3)
	Transgender	1 (0.3)
Age, years		
	18-25	288 (91.4)
	26-35	21 (6.6)
	36-45	5 (1.5)
	46-55	1 (0.5)
Nationality		
	African/African American	20 (6.3)
	Asian/Asian American	25 (7.9)
	Anglo/Caucasian	201 (63.8)
	Hispanic/Latina(o)/Chicana(o)	49 (15.6)
	Alaskan native/native American	2 (0.6)
	Biracial/multiracial	14 (4.4)
	Other	4 (1.3)
Major^a^		
	Health-related^b^	132 (42.9)
	Non health-related^c^	176 (57.1)
Year in college^d^		
	First year	120 (38.1)
	Second year	72 (22.9)
	Third year	65 (20.6)
	Fourth year	56 (17.8)
Exercise		
	Not at all	65 (20.6)
	1-2 times per week	115 (36.5)
	3-4 times per week	70 (22.1)
	5+ times per week	65 (20.6)
Mental health problems		
	Yes	56 (17.5)
	No	259 (82.5)
Health problems		
	Chronic	93 (29.5)
	Acute	5 (1.6)
	None	217 (68.9)

^a^Seven participants did not respond to the item related to their degree.

^b^Health-related majors include degrees related to biomedical sciences, pre-medicine, nursing, etc.

^c^Non health-related majors include engineering, drama, communication, etc.

^d^Two participants did not indicate their year in school.

### Perceived Advantages of Telehealth Systems

Of the students, 295 responded to the advantages item. Thematic analysis of the advantages open-ended item resulted in the generation of 3 overarching themes, as well as several subthemes. The themes that emerged from these responses included accessibility (26.5%, 78/295), convenience (24.4%, 72/295), and efficiency (21.4%, 63/295). Other subthemes from the advantages open-ended item included communication (8.8%, 26/295), connectedness (4.8%, 14/295), affordability (2.4%, 7/295), anonymity (2.4%, 7/295), and usability (2.4%, 7/295). The proportion of themes perceived as advantageous are reported in Table 3.

**Table 3:** Themes related to perceived advantages (N=295) and disadvantages (N=303) of telehealth systems.

Theme		Students, n (%)
Advantages		
	Accessibility	78 (26.5)
	Convenience	72 (24.4)
	Efficiency	63 (21.4)
Advantages subthemes		
	Communication	26 (8.8)
	Connectedness	14 (4.8)
	Affordability	7 (2.4)
	Anonymity	7 (2.4)
	Usability	7 (2.4)
Disadvantages		
	Trust	105 (34.5)
	Impersonality	85 (28.1)
	System errors	59 (19.5)
Disadvantages subthemes		
	Affordability	18 (5.9)
	Communication	7 (2.3)

### Perceived Disadvantages of Telehealth Systems

In total, 303 responses were rated for disadvantages and concerns. Similar themes emerged for responses related to perceived disadvantages, which goes to show that what is perceived as an advantage to some is perceived as a disadvantage to others. That said, the following themes emerged from the disadvantages open-ended item: trust (34.5%, 105/303), impersonality (28.1%, 85/303), and system errors (19.5%, 59/395). Several other subthemes emerged from the disadvantages item including affordability (5.9%, 18/303) and communication (2.3%, 7/303). The proportion of themes perceived as disadvantages are reported in Table 3.

## Discussion

### Principal Findings

It has been well-established in the literature that advantages and disadvantages predict technology adoption, wherein the more advantages a telehealth system has, the more likely individuals are to use the system [11]. The present study utilized a sample of students enrolled in college to support this claim. The thematic analyses indicated that students generally felt as though there were more advantages than disadvantages associated with telehealth systems.

Based on themes derived from this study, students indicated that one major advantage of telehealth systems is that these technologies eliminate many barriers in receiving health care. Many students specifically noted that they would use a telehealth device again in the future if it improved the “availability of services” and “access to these services”. In other words, students could begin to schedule appointments with medical professionals that they perhaps could not normally visit. As well, some students noted that well-designed telehealth systems allow for the “better storage” and “better organization” of health information further facilitating the perceived usability (2.4%, 7/295 of responses) of telehealth technologies.

According to the themes in this study, telehealth devices can “quickly connect” a student to a care provider or practitioner, report health data in “record time”, and “eliminate the need for excessive travel”, all of which are characteristics that exemplify the convenience of telehealth technologies. Many students reported that they would use a telehealth system if it allowed them to conveniently “meet with a doctor at home” and “rapidly connect” to their health information. In a similar vein, many students stated that if a system was efficient, it would allow them to connect to health care services without “wasting time” and “saving money” (ie, these systems can make receiving care more affordable). But, a smaller percentage of students (5.9%, 18/303 of responses) reported the potential cost of telehealth devices to be a disadvantage, particularly if these devices or services were overpriced. Several students specifically stated that if the cost was “too high” they would not use a telehealth device in the future. This is an important factor for engineers and programmers to bear in mind when designing telehealth systems and applications: telehealth systems should be affordable and accessible to all.

In terms of the theme of communication, many students felt that an electronic means of communication would “improve” their relationship with their medical provider because they “could connect with them quickly” and “communicate their concerns in real-time”. Students tended to report that telehealth devices would result in “quicker feedback” from health care providers. Many students felt this would result in “more connectedness” with their health care provider. Only 2.3% (7/303) of student responses indicated that electronic communication via telehealth technology was a perceived disadvantage. On the other hand, almost 3% of the students in our sample noted that one advantage of telehealth systems is that they allow the individual to discuss health issues “more comfortably” or “without feeling embarrassed”. A telehealth device may give more sensitive patients “a protective shield” allowing them to be “more honest” and descriptive about their health concerns. In addition, a few students noted that electronic communication through the use of a telehealth device “can serve as a written record”. For these reasons, students reported that they would likely use a telehealth system in the future to communicate with their health care professional.

While many themes related to the perceived advantages of telehealth systems emerged, many students also pointed out the disadvantages of telehealth systems and indicated that these pitfalls would prevent them from using a telehealth system in the future. One major disadvantage of telehealth systems was user trust. Nearly 35% of students described reservations about using telehealth devices in the future because of issues released to “privacy and security” of personal health information. More specifically, students stated that they would not want their health information to be “given to the wrong person”. Many students suggested that they would not use telehealth systems in the future if there were a “breach of the system” and “personal information was left unprotected”. Almost 2% of students claimed that they would “only trust some systems”, but not all devices, which shows that trust does not always translate from system to system.

Another notable disadvantage was that telehealth devices seem to be “impersonal” and this would result in students being less likely to use telehealth technologies in the future. Importantly, this theme highlights the fact that individuals still want to have “person-to-person interaction”, despite some tradeoffs such as “increased travel time” to the doctor’s office or longer wait times. Many students indicated that “in some cases you just have to see a doctor”. Almost 7% of students stated that they did not want to see “impersonal” telehealth systems fully “replace medical professionals”. As stated by one student, “telehealth might not be as thorough as in-person (visits)”.

Many students voiced concerns about the accuracy or “reliability” of test results that may result due to “system errors”, which is a disadvantage not only for telehealth systems, but for human-computer interaction in general. The theme of system errors tended to overlap with concerns about the potential for “technological malfunctions” and whether or not data would be “saved during a glitch”. To summarize, these perceived disadvantages must be addressed before the adoption of telehealth technologies is widespread within student populations.

Our results also demonstrate partial support for the TAM. For example, one of the most frequently reported disadvantages was trust, which will need to be overcome in order to engage student users with the telehealth device. One way to do this is to convey a clear sense of security over personal health information. Similarly, telehealth devices will have to be well-designed and user friendly, otherwise students may perceive the system as being likely to have system errors or mishandle private health data. Issues with usability, privacy, and security can all effect student perceptions of trust. Perhaps the findings here can be used to conduct more nuanced research on the mechanisms underpinning system trust. In addition, many students touched upon perceived threats, which are defined within the TAM as a fear of technology replacing an occupation. For example, a handful of students explicitly stated that they did not want machines to replace doctors. Many felt that there are serious conditions for which individuals must visit a medical professional.

While our results tended to align with aspects of the TAM framework, the TAM could benefit from the integration of several novel constructs that emerged from the thematic analysis. For example, the TAM does not incorporate factors such as relatedness or connectedness with a medical professional, nor does it address issues of impersonality. At present, the TAM does not incorporate barriers in access to care such as the cost of the device or disease type, which is the strength of the HBM. Given that relatively few students in our sample reported a chronic or acute illness, it is difficult to establish support for the HBM using our student sample.

### Limitations and Future Directions

It could be argued that student populations, typically composed of younger, make less use of health care services because they have a lower incidence of chronic or acute illness. However, with regard to technology adoption and usability, younger adult students may have the fewest barriers in terms of accessing care. Nonetheless student opinions are still important in the assessment of telehealth adoption, especially given that little research exists on student perceptions of telehealth systems. Future studies should aim to better understand how and why students interact with telehealth systems since relatively few studies exist in this domain.

## Abbreviations

HBM: Health Belief Model
PB: behavioral evaluation
PDT: perceived disease threat
PEOU: perceived ease of use
PU: perceived usefulness
TAM: Technology Acceptance Model

## References

[1] American Telemedicine Association (2015). Research Outcomes: Telemedicine's Impact on Healthcare Cost and Quality.

[2] Benavides-Vaello S, Strode A, Sheeran BC (2013). Using technology in the delivery of mental health and substance abuse treatment in rural communities: a review. J Behav Health Serv Res.

[3] Dellifraine JL, Dansky KH (2008). Home-based telehealth: a review and meta-analysis. J Telemed Telecare.

[4] Fischer SH, David D, Crotty BH, Dierks M, Safran C (2014). Acceptance and use of health information technology by community-dwelling elders. Int J Med Inform.

[5] Force S, Brady C (2013). Case study: Lee Memorial Health System the role of telehealth in an integrated health delivery system. Caring.

[6] Malvey D, Slovensky D (2014). mHealth: Transforming Healthcare.

[7] Baker LC, Johnson SJ, Macaulay D, Birnbaum H (2011). Integrated telehealth and care management program for Medicare beneficiaries with chronic disease linked to savings. Health Aff (Millwood).

[8] Cryer L, Shannon SB, Van AM, Leff B (2012). Costs for 'hospital at home' patients were 19 percent lower, with equal or better outcomes compared to similar inpatients. Health Aff (Millwood).

[9] Jackson KM, Scott KE, Graff ZJ, Bateman DA, Flynn JT, Keenan JD, Chiang MF (2008). Cost-utility analysis of telemedicine and ophthalmoscopy for retinopathy of prematurity management. Arch Ophthalmol.

[10] Rojas SV, Gagnon M (2008). A systematic review of the key indicators for assessing telehomecare cost-effectiveness. Telemed J E Health.

[11] Wootton R (2012). Twenty years of telemedicine in chronic disease management--an evidence synthesis. J Telemed Telecare.

[12] Kvedar J, Coye MJ, Everett W (2014). Connected health: a review of technologies and strategies to improve patient care with telemedicine and telehealth. Health Aff (Millwood).

[13] Huang J, Lin S (2009). Exploring the key factors in the choice of home telehealth by using the health belief model. Telemed J E Health.

[14] Davis F, Venkatesh V (1996). A critical assessment of potential measurement biases in the technology acceptance model: three experiments. Int J Hum-Comput St.

[15] Venkatesh V, Davis FD (2000). A theoretical extension of the Technology Acceptance Model: four longitudinal field studies. Manage Sci.

[16] Venkatesh V (2000). Determinants of perceived ease of use: integrating control, intrinsic motivation, and emotion into the Technology Acceptance Model. Inform Syst Res.

[17] Venkatesh V, Thong J (2012). Consumer acceptance and use of information technology: extending the Unified Theory of Acceptance and Use of Technology. MIS Quarterly.

[18] Kuo K, Liu C, Ma C (2013). An investigation of the effect of nurses' technology readiness on the acceptance of mobile electronic medical record systems. BMC Med Inform Decis Mak.

[19] Liu C, Cheng T (2015). Exploring critical factors influencing physicians' acceptance of mobile electronic medical records based on the dual-factor model: a validation in Taiwan. BMC Med Inform Decis Mak.

[20] Gilbert M, Hottes TS, Kerr T, Taylor D, Fairley CK, Lester R, Wong T, Trussler T, Marchand R, Shoveller J, Ogilvie G (2013). Factors associated with intention to use internet-based testing for sexually transmitted infections among men who have sex with men. J Med Internet Res.

[21] Wu K, Zhao Y, Zhu Q, Tan X, Zheng H (2011). A meta-analysis of the impact of trust on technology acceptance model: investigation of moderating influence of subject and context type. Int J Inform Manage.

[22] Glinkowski W, Pawłowska K, Kozłowska L (2013). Telehealth and telenursing perception and knowledge among university students of nursing in Poland. Telemed J E Health.

[23] Braun V, Clarke V (2006). Using thematic analysis in psychology. Qual Res Psychol.

[24] Gale NK, Heath G, Cameron E, Rashid S, Redwood S (2013). Using the framework method for the analysis of qualitative data in multi-disciplinary health research. BMC Med Res Methodol.

[25] Ritchie J, Lewis J, McNaughton Nicholls C (2013). Qualitative Reserach Practice: A Guide for Social Science Students and Researchers.

[26] McHugh ML (2012). Interrater reliability: the kappa statistic. Biochem Med (Zagreb).

[27] Cohen J (1960). A coefficient of agreement for nominal scales. Educ Psychol Meas.

[28] Bull T (2015). Anticipated Telehealth Device Usage in Younger Adults.

